# Insights into the mechanism regulating the differential expression of the P28-OMP outer membrane proteins in obligatory intracellular pathogen *Ehrlichia chaffeensis*

**DOI:** 10.1080/22221751.2021.1899054

**Published:** 2021-03-13

**Authors:** Nan Duan, Xiaohui Ma, Heting Cui, Zhexuan Wang, Zhouyi Chai, Jiaqi Yan, Xiaoxiao Li, Yingxing Feng, Yu Cao, Yongxin Jin, Fang Bai, Weihui Wu, Yasuko Rikihisa, Zhihui Cheng

**Affiliations:** a Key Laboratory of Molecular Microbiology and Technology of the Ministry of Education, Department of Microbiology, College of Life Sciences, Nankai University, Tianjin, People’s Republic of China; b Department of Veterinary Biosciences, College of Veterinary Medicine, The Ohio State University, Columbus, OH, USA

**Keywords:** *Ehrlichia chaffeensis*, human monocytic ehrlichiosis, Tr1, P28-OMP outer membrane proteins, differential gene expression

## Abstract

*Ehrlichia chaffeensis* causes human monocytic ehrlichiosis (HME), which is one of the most prevalent, life-threatening emerging infectious zoonoses. The life cycle of *E. chaffeensis* includes ticks and mammals, in which *E. chaffeensis* proteins are expressed differentially contributing to bacterial survival and infection. Among the *E. chaffeensis* P28-OMP outer membrane proteins, OMP-1B and P28 are predominantly expressed in tick cells and mammalian macrophages, respectively. The mechanisms regulating this differential expression have not been comprehensively studied. Here, we demonstrate that the transcriptional regulators EcxR and Tr1 regulate the differential expression of *omp-1B* and *p28* in *E. chaffeensis.* Recombinant *E. chaffeensis* Tr1 bound to the promoters of *omp-1B* and *p28,* and transactivated *omp-1B* and *p28* promoter-EGFP fusion constructs in *Escherichia coli*. The consensus sequence of Tr1 binding motifs was A^C^/_T_TATA as determined with DNase I footprint assay. Tr1 showed a higher affinity towards the *p28* promoter than the *omp-1B* promoter as determined with surface plasmon resonance. EcxR activated the *tr1* expression in response to a temperature decrease. At 37°C low level of Tr1 activated the *p28* expression. At 25°C high level of Tr1 activated the *omp-1B* expression, while repressing the *p28* expression by binding to an additional site upstream of the *p28* gene. Our data provide insights into a novel mechanism mediated by Tr1 regulating *E. chaffeensis* differential gene expression, which may aid in the development of new therapeutics for HME.

## Introduction

Human monocytic ehrlichiosis (HME) is one of the most prevalent, life-threatening emerging zoonoses. HME is caused by *Ehrlichia chaffeensis*, an obligatory intracellular gram-negative bacterium [1]. The clinical signs of HME include headache, fever, myalgia, anorexia, accompanied by leukopenia, thrombocytopenia, and the elevation of aminotransferases [2]. Cases of HME have been reported in Asia, North America, South America, Europe, and Africa [2]. The number of HME cases reported in the United States was 1799 in 2018, which showed a more than 10-fold increase over a 10-year period [3]. Vaccines are not available for HME. The drug of choice for treating patients is doxycycline, however, HME is a particular threat in elderly and immunocompromised individuals [1].

**Figure 1.**
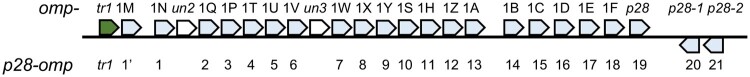
Gene organization of the *E. chaffeensis p28-omp* multigene locus. Genes are represented as boxes with triangles indicating their orientation. The numbers and letters above or below the boxes represent gene IDs.

Differential gene expression in response to the host environment is an important strategy employed by pathogenic bacteria [4]. In *Salmonella enterica* serovar Enteritidis, SEN1538 is regulated by RpoS in response to host and environmental stress, and is critical for bacterial invasion of and replication in macrophages, and systemic infection in mice [5]. *Borrelia burgdorferi,* the tick-borne agent causing Lyme disease, expresses the outer-surface proteins OspA and OspC differentially [6]. OspA is expressed at low temperatures and acts as an adhesin that tethers *B. burgdorferi* to the tick midgut epithelium [7]. OspC is up-regulated at high temperatures and is essential for *B. burgdorferi* infection in mice [8]. The *E. chaffeensis* zoonotic life cycle includes mammalian hosts and tick vectors. *E. chaffeensis* proteins are differentially expressed in mammals and ticks [9–11]. *E. chaffeensis* originating from tick cells persists longer in mice compared to that from canine macrophages, indicating that the differential expression contributes to *E. chaffeensis* transmission and infection [10,12]. P28-OMP outer membrane proteins are highly expressed on the surface of *E. chaffeensis* [1]*.* A β-barrel structure is found in the conserved sequences among these proteins, which functions as a passive diffusion channel to transport sugars and amino acids across the bacterial membrane [13]. P28-OMP outer membrane proteins are encoded by a *p28-omp* multigene locus that contains 22 tandemly arranged paralogous genes (Figure 1) [10,11,14]. P28 (also known as P28-OMP19) is highly expressed by *E. chaffeensis* in mammalian macrophages, whereas OMP-1B (also known as P28-OMP14) is predominantly expressed by *E. chaffeensis* in tick cells [9]. However, the mechanisms regulating the differential expression of the P28-OMP outer membrane proteins in *E. chaffeensis* have not been comprehensively investigated. Northern blotting and primer extension have been used to demonstrate that the genes of *p28* and *omp-1B* are transcribed as monocistronic messages [9–11]. The promoter regions of *omp-1B* and *p28* are bound by *E. chaffeensis* proteins, suggesting that transcriptional regulators are involved in the regulation of differential expression [15]. However, the involved regulators have not been identified. The *tr1* gene, which is upstream of the *p28-omp* multigene locus (Figure 1), encodes a putative transcriptional regulator containing a winged helix-turn-helix motif [1]. It might be feasible that Tr1 regulates the differential expression of the P28-OMP outer membrane proteins, but this possibility has not yet been investigated.

EcxR, ApxR and ErxR are orthologs in *E. chaffeensis*, *Anaplasma phagocytophilum*, and *Ehrlichia ruminantium*, respectively [16–18]. The P44 major surface proteins of *A. phagocytophilum* are also differentially expressed, and contribute to bacterial survival and infection [19,20]. These proteins are mainly expressed from the *p44* expression locus (*p44E*) [1]. ApxR has a regulatory role in the expression of *tr1* and *p44E* [21]. In *E. ruminantium* ErxR is responsible for the coordinated regulation of the expression of *tr1* and *map* genes that are orthologs of the *p28-omp* genes [16]. However, the role of EcxR in regulating the expression of *tr1* and the *p28-omp* genes in *E. chaffeensis* is still unknown.

In the present study, we demonstrated that Tr1 directly binds to the promoters of *omp-1B* and *p28* in *E. chaffeensis.* Furthermore, Tr1 regulates the differential expression of the P28-OMP outer membrane proteins through its expression levels and its affinity towards different binding sites in the promoters of *omp-1B* and *p28.* We also found that the *tr1* expression is activated by EcxR in *E. chaffeensis* in response to a temperature decrease. These findings provide new information on the mechanisms regulating the differential expression of proteins in obligatory intracellular bacteria in various hosts, which may assist in the discovery of next generation HME treatments.

## Materials and methods

### Bacteria and cell culture

*E. chaffeensis* Arkansas strain was propagated in THP-1 cells in RPMI 1640 medium supplemented with 2 mM L-glutamine and 10% fetal bovine serum (Every Green, Zhejiang, China) at 37°C in 5% CO_2_ and 95% air [18].

The bacterial strains used in this study are listed in Table S1. *Escherichia coli* strains DH5α and BL21(DE3) used for general cloning and protein expression, respectively, were cultured in LB supplemented with ampicillin (100 μg/mL) or kanamycin (50 µg/mL), as necessary. Plasmids used in this study are listed in Table S1.

### Expression and purification of recombinant proteins

The *E. chaffeensis tr1* gene was amplified using specific primers (Table S1) and ligated into the pET-33b (+) vector to express recombinant Tr1 (rTr1) with an N-terminal His-tag. The *E. chaffeensis ecxR* gene was ligated into the pET-29a (+) vector to express recombinant EcxR (rEcxR) with a C-terminal His-tag, as described [18]. The ligated plasmids were transformed into *E. coli* DH-5α cells, extracted and confirmed by sequencing. *E. coli* BL21 (DE3) cells were transformed with the plasmids and induced to express rTr1 or rEcxR with 1 mM IPTG at 20°C or 25°C for 5 h, respectively. The recombinant proteins were purified with Ni-affinity chromatography and dialysed against a stocking buffer (10 mM Tris-HCl [pH 7.5], 100 mM NaCl, 1 mM DTT, 10% [v/v] glycerol) [22].

### Electrophoretic mobility shift assay

Electrophoretic mobility shift assay (EMSA) was performed as described with modification [23]. DNA probes were amplified using specific primers (Table S1). To confirm the consensus sequence of Tr1 binding motifs, oligonucleotides were synthesized (GENEWIZ, Suzhou, China) and annealed (Table S1). DNA probes were incubated with rTr1 or rEcxR in a 20 µL reaction (10 mM Tris-HCl [pH 7.5], 100 mM NaCl, 1 mM DTT, 10% [v/v] glycerol) at 25°C for 30 min. Samples were subjected onto an 8% native polyacrylamide gel in 1 × Tris-borate-EDTA (TBE) buffer (44 mM Tris, 44 mM boric acid, 1 mM EDTA, [pH 8.0]) and electrophoresed on ice at 10 mA for 1 h. The gel was stained in 1 × TBE buffer containing 0.5 µg/mL ethidium bromide and visualized with a molecular imager ChemiDoc™ XRS^+^ (Bio-Rad, CA, USA).

For competitive EMSA, rTr1 was incubated with biotinylated DNA probes at 25°C for 30 min. Separate reaction mixtures were prepared with a 50-fold excess of unlabelled DNA competitors. After electrophoresis, DNA probes were transferred to a nylon membrane (Thermo Fisher Scientific, MA, USA) and detected using a Biotin Chromogenic Detection kit (Thermo Fisher Scientific).

### Reporter gene assay

The promoter regions of the target genes were amplified and inserted upstream of the promoter-less *egfp* gene in the pQE60 vector. The DH5α strain containing pBAD harbouring *tr1* (pBAD-rTr1) or *ecxR* (pBAD-rEcxR), or the pBAD vector, was transformed with the EGFP fusion constructs. After inducing the recombinant protein expression with 0.2% (w/v) arabinose at 37°C for 8 h, EGFP fluorescence was measured for each sample using a Varioskan Flash (Thermo Fisher Scientific) (excitation 395 nm, emission 509 nm) and normalized against the OD_600_. The recombinant protein expression was confirmed with Western blotting using an anti-His-tag antibody (Sigma, MO, USA).

### DNase I footprint assay

DNase I footprint assay was performed as described with modification [21]. DNA fragments of the promoters were amplified with specific primers (Table S1). The forward primer was labelled with 6-carboxyfluorescein (6-FAM). The 6-FAM labelled fragment (500 ng) was incubated with 2 or 4 µM rTr1, or 4 µM BSA under the EMSA condition. Then each reaction mixture was added 0.07 U of DNase I (TaKaRa, Dalian, China), and incubated at 25°C for 5 min, then heated at 75°C for 10 min to terminated the digestion. The digested DNA fragments were purified and analysed by GENEWIZ. The sequences of protected regions were analysed using the Peak Scanner software v1.0 (Applied Biosystems, CA, USA).

### Surface plasmon resonance

The affinity of Tr1 towards different DNA fragments was analysed with surface plasmon resonance (SPR) using a Biacore T200 instrument (GE Healthcare, Uppsala, Sweden). In the SPR trials, 120 response units (RU) of biotinylated DNA probes were immobilized by streptavidin on the chip surface. The chip surface was exposed to a serial dilution (2/3 fold) of rTr1 or rEcxR at 25°C for 2 min, then eluted with a running buffer (10 mM Tris-HCl [pH 7.5], 100 mM NaCl, 1 mM DTT, 0.05% Tween 20) at a flow rate of 50 μL/min. The kinetic parameters were analysed with the BIAevaluation software (GE Healthcare) and the final graphs were generated using the GraphPad Prism v5.01 (GraphPad Software, CA, USA).

### Quantitative RT–PCR

*E. chaffeensis* was cultured at different temperatures as described [13]. The infected THP-1 cells were cultured at 37°C for 42 h until small (<1 μm) morulae (microcolonies of bacteria) were detected in more than 80% of the cells. Then half of the culture volume was transferred to 25°C, and the bacterial growth phase was examined by Diff-Quik staining (Leagene, Beijing, China). When the morula size was 1–3 μm in more than 80% of the cells, the total RNA was extracted with BIOZOL Total RNA Extraction Reagent (BioFlux, Beijing, China). The cDNA was synthesized from the total RNA using PrimeScript Reverse Transcriptase (TaKaRa). The cDNA copy numbers of each gene were determined based on the standard curve, which was a 10-fold serial dilution of the pCR II vector harbouring the corresponding DNA fragment. Quantitative real time PCR was performed using specific primers (Table S1) and the SYBR II Green Supermix (Bio-Rad), and the results were analysed using a CFX Connect Real-Time system (Bio-Rad). The expression level of each gene was normalized against *E. chaffeensis 16S rRNA*.

For *E. coli*, total RNA was extracted from each strain after 5 h induction with different concentrations of arabinose. The cDNA copy numbers of *egfp* were determined based on the standard curve prepared as described above. The *egfp* expression level was normalized against *E. coli 16S rRNA*.

## Statistical analysis

Data indicate means ± standard deviations (SD) from three independent experiments performed in triplicate. Statistical analyses were calculated using the Student's *t*-test (two-tailed) and a *P* value <0.05 was considered statistically significant.

## Results

### Tr1 binds to the promoters of omp-1B and p28 and transactivates their expression

**Figure 2.**
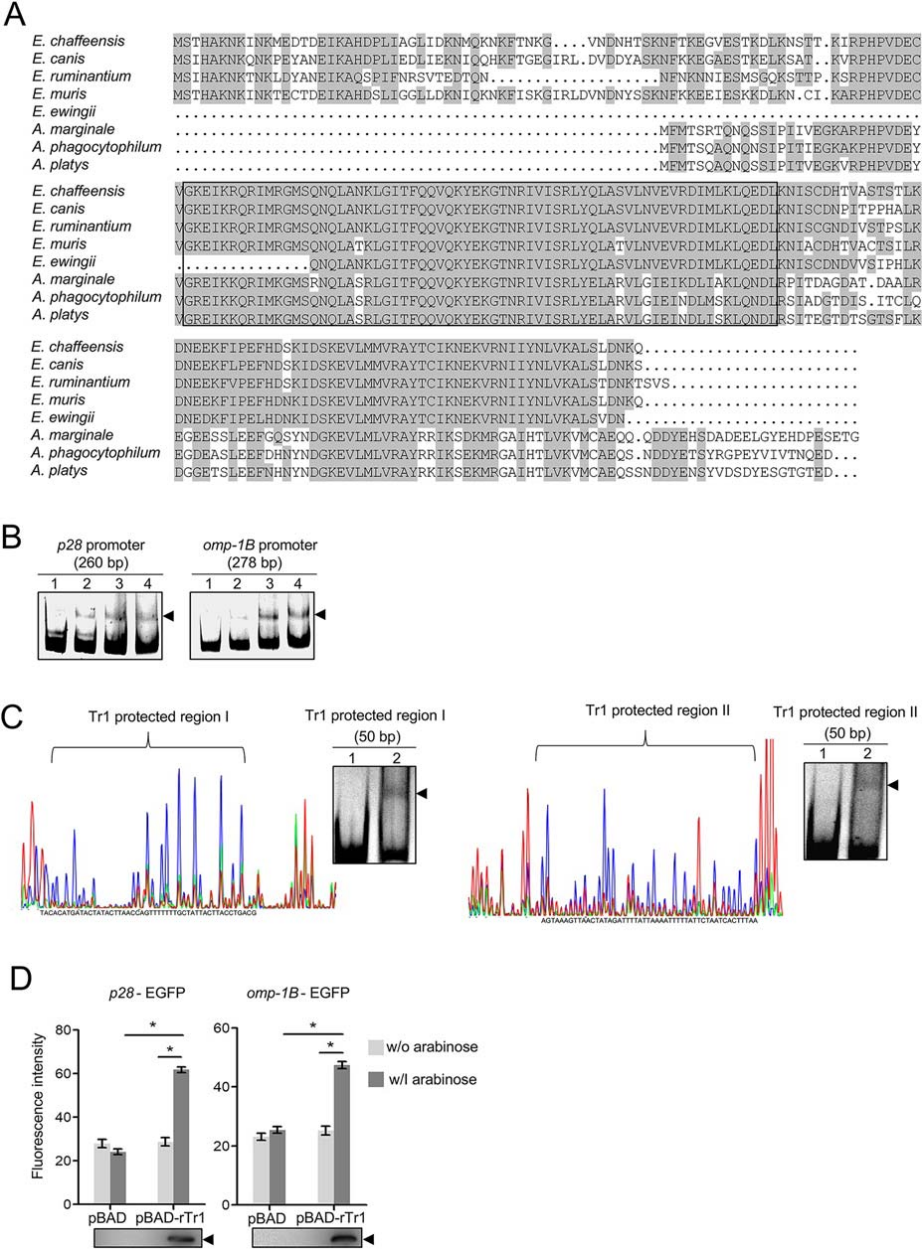
Tr1 regulates the expression of *omp-1B* and *p28*. (A) Comparison of Tr1 orthologs in the genera *Ehrlichia* and *Anaplasma.* Identical amino acids are shaded. The helix-turn-helix DNA-binding motifs are boxed. (B) Tr1 binds to the promoters of *omp-1B* and *p28*. DNA probe (50 ng) was incubated alone (lane 1), or with 0.9, 1.8 or 3.5 µM rTr1 (lane 2–4). Shifted bands are indicated by arrowheads. (C) Identification of the sequences protected by Tr1 in the promoters of *p28* and *omp-1B*. Left, electropherograms are superimposed to show the region protected by different concentrations of rTr1 (green, 2 µM; red, 4 µM) or BSA (blue, 4 µM) within the *p28* promoter or *omp-1B* promoter. Right, annealed DNA probe (150 ng) was incubated alone (lane 1), or with 1.8 µM rTr1 (lane 2). Shifted bands are indicated by arrowheads. (D) Tr1 activates the expression of *omp-1B* and *p28*. DH5α strains were induced with arabinose. The fluorescence intensity was normalized against the OD_600_ of each strain. Data indicate means ± SD. *, *P* < 0.05 determined with the Student's *t*-test. Western blotting shows the rTr1 expression.

We first compared the sequences of Tr1 orthologs in the genera *Ehrlichia* and *Anaplasma*. *E. chaffeensis* Tr1 shows higher than 70% amino acid sequence identity to Tr1 orthologs in *E. ruminantium*, *Ehrlichia canis* and *Ehrlichia muris*. The sequence of *Ehrlichia ewingii* Tr1 is only partially available, which is highly conserved to the corresponding part of *E. chaffeensis* Tr1 (Figure 2A). Whereas *E. chaffeensis* Tr1 shows 39–40% amino acid sequence identity to Tr1 orthologs in *A. phagocytophilum*, *Anaplasma marginale* and *Anaplasma platys* (Figure 2A). The helix-turn-helix DNA binding motifs are highly conserved in all orthologs compared (Figure 2A). These data suggest that the function of Tr1 might be conserved in the genus *Ehrlichia,* and that Tr1 recognizes and binds to the same DNA sequence in the genera *Ehrlichia* and *Anaplasma*.

We then expressed and purified *E. chaffeensis* rTr1 from *E. coli.* The purified rTr1 showed a single band in the SDS-PAGE gel (Fig. S1). The DNA probes derived from the promoters of *omp-1B* and *p28* were shifted upon incubation with rTr1 (Figure 2B), indicating that Tr1 directly binds to the promoters of *omp-1B* and *p28.* The Tr1 binding sites in these promoters were determined using DNase I footprint assay. The protected regions of rTr1 ranged from −143 to −94 in the *p28* promoter (Tr1 protected region I) and from −130 to −81 in the *omp-1B* promoter (Tr1 protected region II) calculated from the transcriptional start sites [10], respectively (Figure 2C). The binding of rTr1 to these regions was confirmed with EMSA (Figure 2C).

Due to the lack of genetic manipulation tools to study *E. chaffeensis* gene function, we examined whether Tr1 transactivates the expression of *omp-1B* and *p28* in *E. coli.* The promoter region of *omp-1B* (278 bp) or *p28* (260 bp) was inserted upstream of the promoter-less *egfp* gene in the pQE60 vector to generate *omp-1B*-EGFP or *p28*-EGFP fusion construct (Fig. S2). The DH5α strain containing the pBAD vector harbouring *E. chaffeensis tr1* gene (pBAD-rTr1) or the pBAD vector (negative control) was transformed with the EGFP fusion constructs, respectively. The rTr1 expression induced with arabinose resulted in a significant increase in EGFP fluorescence intensity in bacteria harbouring *omp-1B*-EGFP or *p28*-EGFP compared to no induction or the vector controls (Figure 2D). These results indicate that Tr1 activates the expression of *omp-1B* and *p28*.

### The tr1 expression is autoregulated

**Figure 3.**
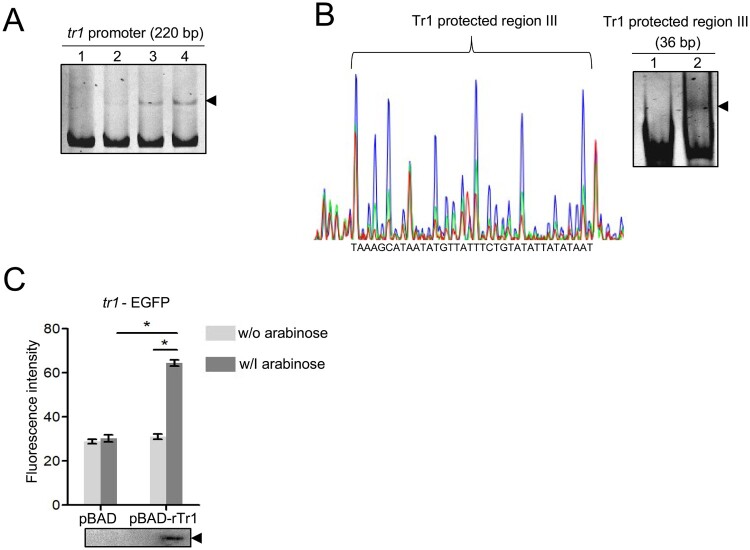
The *tr1* expression is autoregulated. (A) Tr1 binds to the *tr1* promoter. DNA probe (50 ng) was incubated alone (lane 1), or with 0.9, 1.8 or 3.5 µM rTr1 (lane 2–4). Shifted bands are indicated by an arrowhead. (B) Identification of the sequence protected by Tr1 in the *tr1* promoter. Left, electropherograms are superimposed to show the region protected by different concentrations of rTr1 (green, 2 µM; red, 4 µM) or BSA (blue, 4 µM) within the *tr1* promoter. Right, annealed DNA probe (150 ng) was incubated alone (lane 1) or with 1.8 µM rTr1 (lane 2). The shifted band is indicated by an arrowhead. (C) Tr1 activates the *tr1* expression. DH5α strains were induced with arabinose. The fluorescence intensity was normalized against the OD_600_ of each strain. Data indicate means ± SD. *, *P* < 0.05 determined with the Student's *t*-test. Western blotting shows the rTr1 expression.

Since many regulators regulate their own expression, we examined whether Tr1 binds to its own promoter. The DNA probe derived from the *tr1* promoter was shifted upon incubation with rTr1 (Figure 3A). The DNase I footprint assay showed that the region protected by Tr1 ranged from −144 to −109 calculated from the translational start codon of the *tr1* gene (Tr1 protected region III) (Figure 3B). The binding of rTr1 to this region was confirmed with EMSA (Figure 3B).

Next, we examined whether Tr1 transactivates its own expression. The *tr1*-EGFP fusion construct was generated by inserting the *tr1* promoter (220 bp) upstream of the promoter-less *egfp* gene in the pQE60 vector. The DH5α strain containing pBAD-rTr1 or pBAD was transformed with *tr1*-EGFP. The rTr1 expression induced with arabinose resulted in a significant increase in EGFP fluorescence intensity compared to the controls (Figure 3C). These data indicate that the *tr1* expression is positively autoregulated in *E. chaffeensis*.

### The consensus sequence of Tr1 binding motifs is A^C^/_T_TATA

**Figure 4.**
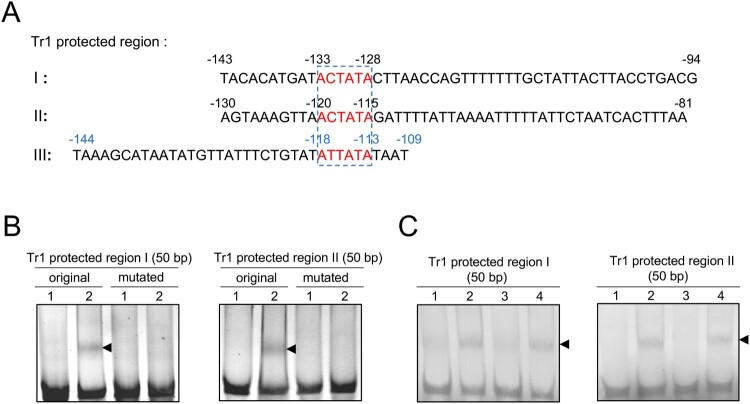
The consensus sequence of Tr1 binding motifs is A^C^/_T_TATA. (A) The sequences protected by Tr1 in the promoter regions of *p28*, *omp-1B* and *tr1* are aligned. The numbers in black or blue indicate the position calculated from the transcriptional or translational start sites, respectively. The predicted consensus sequences of Tr1 binding motifs are boxed. (B) The annealed Tr1 protected region I or II (original or mutated) (150 ng) was incubated alone (lane 1), or with 1.8 µM rTr1 (lane 2). (C) The biotinylated Tr1 protected region I or II (100 ng) was incubated alone (lane 1), with 1 µM rTr1 (lane 2), or with rTr1 plus 50-fold excess of the corresponding unlabelled region I or II (lane 3), or mutated unlabelled region I or II (lane 4). Shifted bands are indicated by arrowheads.

Since the consensus sequence of Tr1 binding motifs has not been determined, we aligned the sequences of the Tr1 protected regions and predicted A^C^/_T_TATA is the consensus sequence of Tr1 binding motifs (Figure 4A). To confirm this, we mutated ACTATA in the Tr1 protected region I and II to GGGGGG, then incubated the mutated DNA fragments with rTr1. No shifted band was detected (Figure 4B). Competitive EMSA was performed for further confirmation. The band shifting of the biotinylated probes of the Tr1 protected region I and II upon incubation with rTr1 was abolished when 50-fold of the corresponding unlabelled competitor was added. However, 50-fold of the mutated unlabelled competitors showed no effect on the band shifting (Figure 4C). These data indicate that A^C^/_T_TATA is the consensus sequence of Tr1 binding motifs.

### The tr1 expression is regulated by EcxR

**Figure 5.**
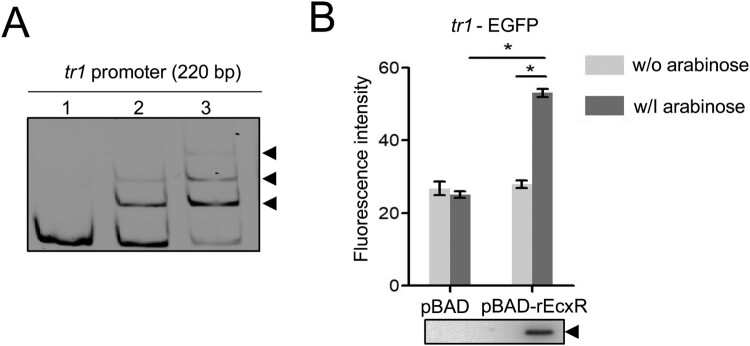
The *tr1* expression is regulated by EcxR. (A) EcxR binds to the *tr1* promoter. DNA probe (50 ng) was incubated alone (lane 1), or with 1.8 or 3.5 µM rEcxR (lane 2, 3). Shifted bands are indicated by arrowheads. (B) EcxR activates the *tr1* expression. DH5α strains were induced with arabinose. The fluorescence intensity was normalized against the OD_600_ of each strain. Data indicate means ± SD. *, *P* < 0.05 determined with the Student's *t*-test. Western blotting shows the rEcxR expression.

We next investigated whether the *tr1* expression is regulated by EcxR. The purified rEcxR showed a single band in the SDS-PAGE gel (Fig. S3A). Shifted bands were detected when rEcxR was incubated with the *tr1* promoter (Figure 5A). We then examined whether EcxR activates the *tr1* expression*.* The DH5α strain containing pBAD harbouring the *ecxR* gene (pBAD-rEcxR) or pBAD was transformed with *tr1*-EGFP. The rEcxR expression induced with arabinose resulted in a significant increase in EGFP fluorescence intensity compared to the controls (Figure 5B). These data indicate that the *tr1* expression is regulated by EcxR in *E. chaffeensis*. Shifted bands were detected when rEcxR was incubated with the promoters of *omp-1B* and *p28* (Fig. S3B), indicating that EcxR may also play a role in the regulation of *omp-1B* and *p28*.

### Low temperature up-regulates the expression of ecxR and tr1 leading to the differential expression of omp-1B and p28

**Figure 6.**
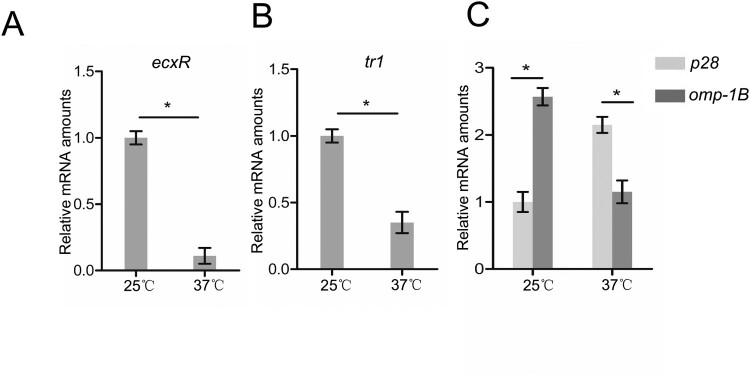
The expression of *ecxR*, *tr1*, *p28* and *omp-1B* in *E. chaffeensis* at 25°C or 37°C. RNA samples were prepared from *E. chaffeensis*-infected THP-1 cells cultured at 25°C or 37°C. The expression levels of *ecxR* (A), *tr1* (B), *p28* and *omp-1B* (C) at different temperatures were normalized against *E. chaffeensis 16S rRNA*. The value of *ecxR*, *tr1* or *p28* at 25°C was set as 1, respectively. Data indicate means ± SD. *, *P* < 0.05 determined with the Student's *t*-test.

Mammals and ticks have different body temperatures. When cultured in canine macrophage DH82 cells, different temperatures cause the differential expression of *E. canis p30–10* and *p30–1*, the orthologs of *E. chaffeensis omp-1B* and *p28,* respectively [24,25]. We then examined the expression of *ecxR*, *tr1*, *omp-1B* and *p28* in *E. chaffeensis* cultured in human macrophage THP-1 cells at different temperatures. The expression of *ecxR* and *tr1* was significantly higher in *E. chaffeensis* cultured at 25°C than that at 37°C (Figure 6A and B). The *omp-1B* expression was higher at 25°C than that at 37°C, whereas the *p28* expression pattern was opposite (Figure 6C). These data suggest that the up-regulation of *ecxR* at low temperatures activates the *tr1* expression contributing to the differential expression of *omp-1B* and *p28*.

### Tr1 regulates the differential expression of omp-1B and p28

**Figure 7.**
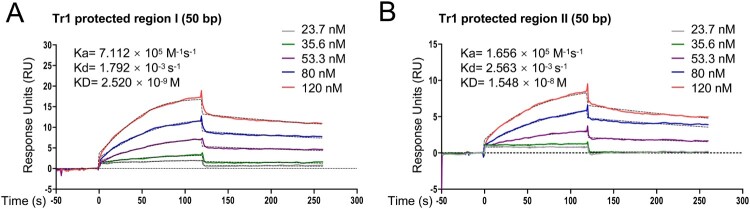
Tr1 has different affinities towards the promoters of *p28* and *omp-1B*. The data indicate SPR determination of interplay between rTr1 and the Tr1 protected region I in the *p28* promoter (A) or the Tr1 protected region II in the *omp-1B* promoter (B). *k_a_*, association rate constant; *k_d_*, dissociation rate constant; *K_D_*, the equilibrium dissociation constant. The concentrations of the proteins are indicated in the inset of the figures.

Since Tr1 is differentially expressed at 25°C and 37°C, we hypothesized that the Tr1 amount may contribute to the differential expression of *omp-1B* and *p28.* Then we investigated the affinity of Tr1 towards the promoters of *omp-1B* and *p28*. We performed SPR using the Tr1 protected regions and found that Tr1 had different binding affinities towards these regions*.* The *K_D_* of rTr1 to the Tr1 protected region II (in the *omp-1B* promoter) was 1.548×10^−8^ M, which was 6.1 times higher than the *K_D_* of rTr1 to the Tr1 protected region I (in the *p28* promoter), 2.520×10^−9^ M (Figure 7A and B), indicating that Tr1 has a higher affinity towards the *p28* promoter than the *omp-1B* promoter. No binding was detected when rEcxR was exposed to these two regions (Fig. S4), suggesting that EcxR may bind to different sites in the promoters of *omp-1B* and *p28*. Considering the expression patterns of *ecxR*, *tr1, p28* and *omp-1B* at different temperatures (Figure 6), our results suggest that the *p28* promoter with a higher affinity is bound and activated by Tr1, when the Tr1 amount in *E. chaffeensis* is low at 37°C, whereas at 25°C the Tr1 amount in *E. chaffeensis* is high enough to activate the *omp-1B* promoter with the lower affinity.

**Figure 8.**
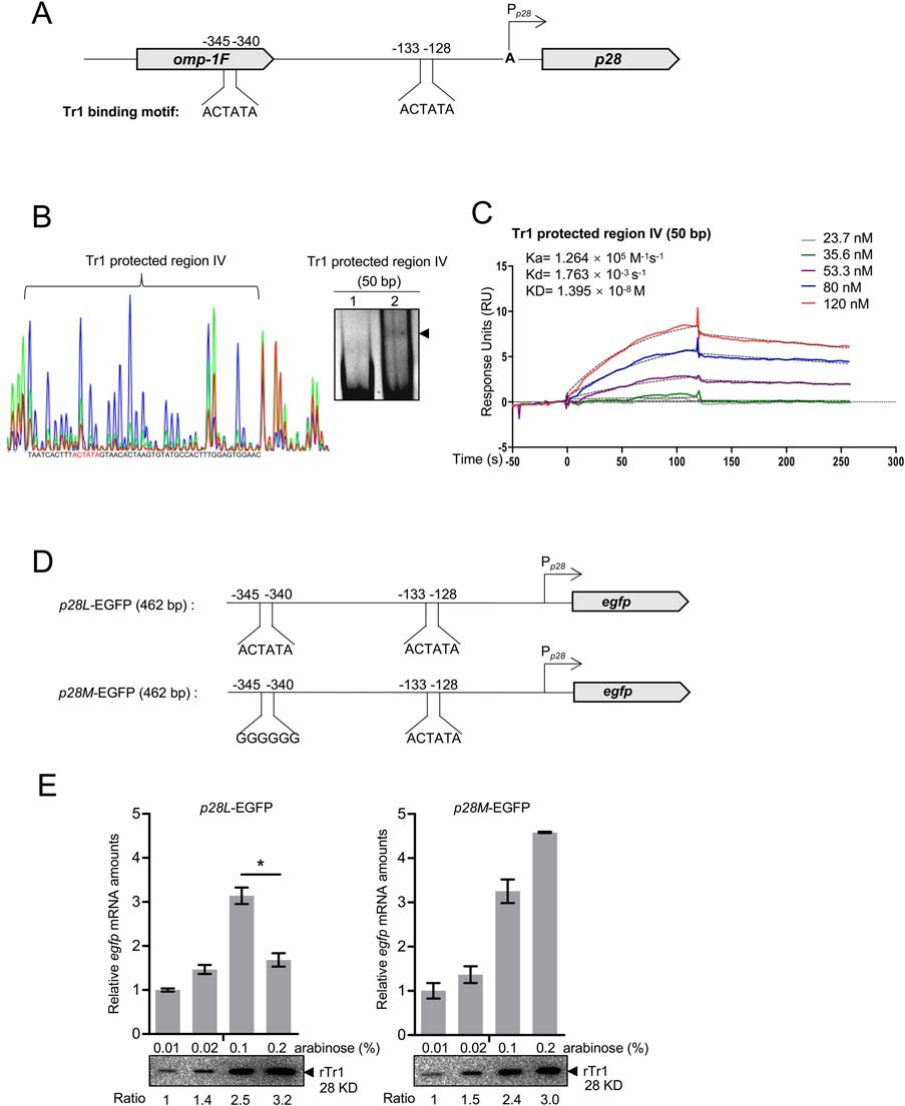
Tr1 binds to an additional binding site to repress the *p28* expression. (A) Diagram of the Tr1 binding motifs located upstream of the *p28* gene. The numbers indicate the position calculated from the transcriptional start site. (B) Identification of the sequence of the additional Tr1 binding site. Left, electropherograms are superimposed to show the region protected by different concentrations of rTr1 (green, 2 µM; red, 4 µM) or BSA (blue, 4 µM). The red letters indicate Tr1 binding motif. Right, annealed DNA probe (150 ng) was incubated alone (lane 1), or with 1.8 µM rTr1 (lane 2). The shifted band is indicated by an arrowhead. (C) The rTr1 affinity towards the Tr1 protected region IV. The data indicate SPR determination of interplay between rTr1 and the Tr1 protected region IV. *k_a_*, association rate constant; *k_d_*, dissociation rate constant; *K_D_*, the equilibrium dissociation constant. The concentrations of the proteins are indicated in the inset of the figure. (D) Diagram of the fusion constructs. The name and the inserted-fragment length are shown on the left. (E) Tr1 represses the *p28* expression with the presence of Tr1 protected region IV. DH5α strains were induced with different concentrations of arabinose. The *egfp* expression level of each sample was normalized against *E. coli 16S rRNA*. Data indicate means ± SD. *, *P* < 0.05 determined with the Student's *t*-test. Western blotting was performed to determine the rTr1 amount in each sample, which was normalized against the value of OD_600_. The numbers below the panels indicate the relative values of the intensity of each band to that of 0.01% arabinose induction.

Then we investigated the mechanism by which the *p28* expression is repressed at 25°C. Many transcription factors repress downstream gene expression by binding to an additional binding site and forming a DNA loop [26,27]. We screened the upstream regions of *omp-1B* and *p28* up to −500 bp from the transcriptional start sites, and found an additional Tr1 binding motif upstream of the *p28* start site (−345 to −340) (Figure 8A), which is located in the encoding region of the *omp-1F* gene, but none in the upstream region of *omp-1B*. The DNase I footprint assay determined that the protected region was from −306 to −355 (Tr1 protected region IV), which includes the binding motif (Figure 8B). EMSA results confirmed the Tr1 binding to this region (Figure 8B). SPR showed that the *K_D_* of rTr1 to this region was 1.395×10^−8^ M (Figure 8C), which was higher than that of the Tr1 protected region I, but at the same level of the Tr1 protected region II. Then we investigated whether this region is involved in the regulation of *p28* expression. We generated a *p28L*-EGFP fusion construct by inserting a 462-bp fragment derived from the *p28* promoter region including the Tr1 protected region I and IV upstream of the promoter-less *egfp* gene in the pQE60 vector, and a *p28M*-EGFP fusion construct by mutating the Tr1 binding motif within the Tr1 protected region IV in the *p28L*-EGFP construct to GGGGGG (Figure 8D). The *p28L*-EGFP or *p28M*-EGFP construct was transformed into the DH5α strain harbouring pBAD-rTr1, respectively. Since the EGFP protein is stable, we examined the *egfp* mRNA levels and rTr1 amounts in these two resulting strains induced with different concentrations of arabinose for 5 h. As the rTr1 amount increased, the *egfp* mRNA level increased in the strain harbouring *p28M*-EGFP (Figure 8E). However, the *egfp* mRNA level decreased in the strain harbouring *p28L*-EGFP with 0.2% arabinose induction, which showed the highest rTr1 amount (Figure 8E). The copy numbers of the plasmids were at the same level in all samples (Fig. S5). All these data suggest that with the increase in the Tr1 amount, Tr1 binds to an additional binding site in the *p28* promoter and represses the *p28* expression.

We also examined the *egfp* mRNA levels and the rTr1 amounts in the strains harbouring *p28*-EGFP or *omp-1B*-EGFP. As the rTr1 amount increased, the *egfp* mRNA levels increased in the strain harbouring *p28*-EGFP comparable to those in the strain harbouring *p28M*-EGFP. The *egfp* up-regulation in the strain harbouring *omp-1B*-EGFP was lower and required higher amount of rTr1 than that in the strain harbouring *p28*-EGFP (Fig. S6).

## Discussion

By coordinately altering gene expression, pathogenic bacteria respond to their environments and facilitate their transmission and establishment of infections in different hosts. *E. chaffeensis* expresses genes differentially for rapid adaptation when transmitted from ticks to mammals [9]. Here, we demonstrated that *E. chaffeensis* Tr1 is a functional regulator and plays an important role in regulating the differential expression of outer membrane proteins. Since Tr1 orthologs are conserved among the genera *Ehrlichia* and *Anaplasma*, understanding the role of Tr1 in host adaptation and gene expression regulation will lead to novel prophylactic and therapeutic targets to prevent and cure infections caused by these bacteria.

Previous results showed that the genes of *p28* and *omp-1B* are transcribed predominantly by σ^70^, and the −35 motifs and the AT-rich spacers between −10 and −35 motifs in the promoters of these genes contribute to the differential expression [28,29]. We found that the Tr1 binding affinities towards these promoters were different. The Tr1 protected region I in the *p28* promoter showed a much higher affinity towards Tr1 compared to the Tr1 protected region II in the *omp-1B* promoter. The body temperature of mammals is higher than that of ticks, and the *tr1* expression is relatively low in *E. chaffeensis* at high temperatures. Thus the Tr1 amount is low in *E. chaffeensis* growing in mammals and the *p28* promoter with the higher affinity is bound and activated. When *E. chaffeensis* grows in ticks, low temperature up-regulates the *tr1* expression via EcxR. The large amount of Tr1 binds to the *omp-1B* promoter with a lower affinity and activates its expression. The Tr1 protected region IV upstream of the *p28* gene showed a weaker affinity towards Tr1 than the Tr1 protected region I. When the Tr1 amount increases at low temperature, Tr1 binds to this region and represses the *p28* expression. Like other transcriptional regulators [26,27], Tr1 may also form a DNA loop by dimerization to repress the *p28* expression. This possibility remains to be investigated.

We found that *E. chaffeensis* EcxR bound to the promoters of *omp-1B* and *p28*, but the binding sites were different from those of Tr1. It is likely that the expression of *omp-1B* and *p28* is under the control of a complex regulatory network. EcxR regulates their expression upon a temperature shift and the autoregulation of Tr1 amplifies the signal leading to a robust response. *E. chaffeensis* EcxR also regulates the expression of the type IV secretion system (TFSS) [18]. Thus the expression of outer membrane proteins and TFSS might be coordinated by EcxR for *E*. *chaffeensis* to adapt to various hosts.

We determined that the consensus sequence of Tr1 binding motifs is A^C^/_T_TATA. Due to the reduction of the bacterial genome during evolution, *E. chaffeensis* has only a few transcriptional regulators in its genome, which results in merge of genes especially virulent genes into the regulons of these regulators. NtrX regulates the expression of *putA* and *glnA*, which are important for *E. chaffeensis* to initiate and establish infection [30]. CtrA regulates the expression of *bolA*, *ompA*, and *surE*, which are important for *E. chaffeensis* infection and intracellular survival [31]. The helix-turn-helix DNA-binding motifs in Tr1 of the genera *Ehrlichia* and *Anaplasma* are highly conserved, thus Tr1 in these bacteria may recognize the same binding motif. The consensus sequence of *E. chaffeensis* Tr1 binding motifs may be helpful to screen for more genes regulated by Tr1 and to understand how *Ehrlichia* and *Anaplasma* spp. harness gene expression for the adaptation to different hosts.

## Supplementary Material

supplementary_table-word_version_.docx

fig_s6.tif

fig_s5.tif

fig_s4.tif

fig_s3.tif

fig_s2.tif

fig_s1.tif
