# Supplementary material for: Insights into the mechanism regulating the differential expression of the P28-OMP outer membrane proteins in obligatory intracellular pathogen Ehrlichia chaffeensis
Source: Emerg Microbes Infect. 2021 Mar 13;10(1):461–71. doi: 10.1080/22221751.2021.1899054 (PMC7971322; doi:10.1080/22221751.2021.1899054)
Supplement: supplementary_table-word_version_.docx [file TEMI_A_1899054_SM8602.docx]

**Supplemental material**

**Table S1. Bacterial strains, plasmids and primers used in this study.**

| **Strain/ Plasmid /Primer** | **Description** | **Source (Reference)** |
| --- | --- | --- |
| 1. ***coli*** | | |
| BL21(DE3)/Tr1 | BL21(DE3) expressing Tr1; Kan^r^ | This study |
| BL21(DE3)/EcxR | BL21(DE3) expressing EcxR; Kan^r^ | This study |
| DH5α/pBAD-rTr1 | DH5α expressing Tr1; Kan^r^ | This study |
| DH5α/pBAD-rEcxR | DH5α expressing EcxR; Kan^r^ | This study |
| DH5α/pQE60-*omp-1B* | DH5α / pQE60 containing promoter *omp-1B*; Amp^r^ | This study |
| DH5α/pQE60-*p28* | DH5α / pQE60 containing promoter *p28*; Amp^r^ | This study |
| DH5α/pQE60-*tr1* | DH5α / pQE60 containing promoter *tr1*; Amp^r^ | This study |
| DH5α/pBAD/*omp-1B* | DH5α harbouring pBAD and pQE60-*omp-1B* plasmids; Kan^r^, Amp^r^ | This study |
| DH5α/pBAD/*p28* | DH5α harbouring pBAD and pQE60-*p28* plasmids; Kan^r^, Amp^r^ | This study |
| DH5α/pBAD/*tr1* | DH5α harbouring pBAD and pQE60-*tr1* plasmids; Kan^r^, Amp^r^ | This study |
| DH5α/pBAD-rTr1/*omp-1B* | DH5α harbouring pBAD-rTr1 and pQE60-*omp-1B* plasmids; Kan^r^, Amp^r^ | This study |
| DH5α/pBAD-rTr1/*p28* | DH5α harbouring pBAD-rTr1 and pQE60-*p28* plasmids; Kan^r^, Amp^r^ | This study |
| DH5α/pBAD-rTr1/*tr1* | DH5α harbouring pBAD-rTr1 and pQE60*-tr1* plasmids; Kan^r^, Amp^r^ | This study |
| DH5α/pBAD-rEcxR/*tr1* | DH5α harbouring pBAD-rEcxR and pQE60-*tr1* plasmids; Kan^r^, Amp^r^ | This study |
| **Plasmid** |  |  |
| pET-33b(+) | Clone vector; Kan^r^ | Novagen |
| pET-29a(+) | Clone vector; Kan^r^ | Novagen |
| pTr1 | pET-33b(+) harbouring *tr1* gene; Kan^r^ | This study |
| pEcxR | pET-29a(+) harbouring *ecxR* gene; Kan^r^ | This study |
| pBAD | Clone vector; Kan^r^ | From Doctor Bi |
| pBAD-rTr1 | pBAD harbouring *tr1* gene; Kan^r^ | This study |
| pBAD-rEcxR | pBAD harbouring *ecxR* gene; Kan^r^ | This study |
| pQE60-EGFP  pQE60-*tr1*  pQE60-*omp-1B*  pQE60-*p28* | Amp^r^  pQE60 harbouring *tr1* promoter; Amp^r^  pQE60 harbouring *omp-1B* promoter; Amp^r^  pQE60 harbouring *p28* promoter; Amp^r^ | From Doctor Bi  This study  This study  This study |
| **Primer** | **Sequence (5’→3’)** | **Function** |
| Tr1-F | GGGGGATCCGATGTCTACACATGCGAAAAACA | Protein expression |
| Tr1-R | GGGAAGCTTTTACTGTTTGTTATCTAAAGACAA | Protein expression |
| EcxR-F | TATACATATGACAACAATAAGTAACCAAAATG | Protein expression |
| EcxR-R | GTGCTCGAGATCTTCTTTTTGTATTATTACAAGA | Protein expression |
| pBAD-rTr1-F | CCCCATATGGATGTCTACACATGCGAAAAACA | EGFP reporter assay |
| pBAD-rTr1-R | CCCGGATCCTTACTGTTTGTTATCTAAAGACAA | EGFP reporter assay |
| pBAD-rEcxR-F | CCCCATATGATGACAACAATAAGTAACCAAAAT | EGFP reporter assay |
| pBAD-rEcxR-R | CCCGGATCCTTAATCTTCTTTTTGTATTATTACAA | EGFP reporter assay |
| pQE60-*omp-1B*-F | GGGCTCGAGGAATTTGTGATTTGAAATAACAAG | EGFP reporter assay |
| pQE60-*omp-1B*-R | CCCGAATTCGTTAATAAACCTTTTATAAAAGATAA | EGFP reporter assay |
| pQE60-*p28*-F | GGGCTCGAGtgctgcaggtaaataaaaatagt | EGFP reporter assay |
| pQE60-*p28-*R | CCCGAATTCATATAACCTAATAGTGACAAATAAA | EGFP reporter assay |
| pQE60-*tr1-*F | GGGCTCGAGACCCGGACAAACTGACTTTC | EGFP reporter assay |
| pQE60-*tr1-*R | CCCGAATTCCCTATAACAAAGCAACCTATGT | EGFP reporter assay |
| pQE60-*p28L*-F | GGGCTCGAGGatattcctgctatgataccc | EGFP reporter assay |
| pQE60-*p28L*-R | CCCGAATTCATGATATCAATGCACTTGTTATG | EGFP reporter assay |
| pQE60-*p28M*-F | TTAGTGTTACCCCCCCAAAGTGATTACCTGT | EGFP reporter assay |
| pQE60-*p28M*-R | GGGGGGgtaacactaagtgtat | EGFP reporter assay |
| *tr1*-F^b^ | ACCCGGACAAACTGACTTTC | EMSA and DNase I footprint assay |
| *tr1*-R | CCTATAACAAAGCAACCTATGT | EMSA and DNase I footprint assay |
| *omp-1B*-F | GAATTTGTGATTTGAAATAACAAG | EMSA |
| *omp-1B*-R | GTTAATAAACCTTTTATAAAAGATAA | EMSA |
| *p28*-F | Tgctgcaggtaaataaaaatagt | EMSA |
| *p28*-R | ATATAACCTAATAGTGACAAATAAA | EMSA |
| Tr1 protected region I-F^a,c^ | tacacatgatactatacttaaccagtttttttgctattacttacctgacg | EMSA and SPR |
| Tr1 protected region I-R^a^ | CGTCAGGTAAGTAATAGCAAAAAAACTGGTTAAGTATAGTATCATGT | EMSA and SPR |
| Tr1 protected region Ⅱ-F^a,c^ | AGTAAAGTTAACTATAGATTTTATTAAAATTTTTATTCTAATCACTTTAA | EMSA and SPR |
| Tr1 protected region Ⅱ-R^a^ | TTAAAGTGATTAGAATAAAAATTTTAATAAAATCTATAGTTAACTTTACT | EMSA and SPR |
| Tr1 protected region I with mutated binding motif-F^a^ | tacacatgatGGGGGGcttaaccagtttttttgctattacttacctgacg | EMSA |
| Tr1 protected region I with mutated binding motif-R^a^ | CGTCAGGTAAGTAATAGCAAAAAAACTGGTTAAGCCCCCCATCATGTGTA | EMSA |
| Tr1 protected region Ⅱ with mutated binding motif-F^a^ | AGTAAAGTTAGGGGGGGATTTTATTAAAATTTTTATTCTAATCACTTTAA | EMSA |
| Tr1 protected region Ⅱ with mutated binding motif-R^a^ | TTAAAGTGATTAGAATAAAAATTTTAATAAAATCCCCCCCTAACTTTACT | EMSA |
| Tr1 protected region III-F^a^ | TAAAGCATAATATGTTATTTCTGTATATTATATAAT | EMSA |
| Tr1 protected region III-R^a^ | ATTATATAATATACAGAAATAACATATTATGCTTTA | EMSA |
| Tr1 protected region IV-F^a,c^ | Taatcactttactatagtaacactaagtgtatgccactttggagtggaac | EMSA and SPR |
| Tr1 protected region IV-R ^a^ | GTTCCACTCCAAAGTGGCATACACTTAGTGTTACTATAGTAAAGTGATTA | EMSA and SPR |
| *omp-1B*-F^b^ | GAATTTGTGATTTGAAATAACAAG | DNase I footprint assay |
| *omp-1B*-R | GTTAATAAACCTTTTATAAAAGATAA | DNase I footprint assay |
| *p28*-F^b^ | TATAAGTGCTGTTTTTCTCACCTTTA | DNase I footprint assay |
| *p28*-R | ATGATATCAATGCACTTGTTATG | DNase I footprint assay |
| *p28* Ⅱ-F^b,d^ | gtgatagggaatgaattcagag | DNase I footprint assay |
| *p28* Ⅱ-R^d^ | TAAAGGTGAGAAAAACAGCACTTATA | DNase I footprint assay |
| *16S rRNA*-F (*E. chaffeensis*) | GGTGAGTAATGCGTAGGAATC | RT-PCR |
| *16S rRNA*-R (*E. chaffeensis*) | GCTCATCTAATAGCGATAAATC | RT-PCR |
| *16S rRNA*-F (*E. coli*) | GCGGTAATACGGAGGGTGC | RT-PCR |
| *16S rRNA*-R (*E. coli*) | TGAGCGTCAGTCTTCGTCCA | RT-PCR |
| *tr1-*RT-F | TTGCAAGCGTACTTAACGTTG | RT-PCR |
| *tr1-*RT-R | TTTGCTATCGTGAAACTCTGG | RT-PCR |
| *omp-1B*-RT-F | TCTCAGGAAGTATTGGTTATG | RT-PCR |
| *omp-1B*-RT-R | CCAAAGTATTTATAGTAGTCAC | RT-PCR |
| *ecxR*-RT-F | TGGTAAAACAGCAGGATTTTCA | RT-PCR |
| *ecxR*-RT-R | CTAGGGAACTCTCTGCCATA | RT-PCR |
| *p28*-RT-F | TACTCAATGGATGGTCCAAG | RT-PCR |
| *p28*-RT-R | TCTGCTGCTGAGTTATGGG | RT-PCR |
| *egfp*-RT-F | ATACCCAGATCATATGAAGCG | RT-PCR |
| *egfp*-RT-R | TTGTATTCCAACTGTTGGCC | RT-PCR |

The enzymes sites are indicated by the underline.

Kan^r^, kanamycin resistance; Amp^r^, ampicillin resistance.

^a^This set of oligonucleotides was annealed for EMSA.

^b^This primer was also labelled with 6-FAM and used to amplify the fragment for DNase I footprint assay.

^c^This primer was also biotinylated and used for SPR and competitive EMSA.

^d^This primer was used to amplify the fragment for DNase I footprint assay for Tr1 protected region IV.
