# Supplementary figures and images for: Insights into the mechanism regulating the differential expression of the P28-OMP outer membrane proteins in obligatory intracellular pathogen Ehrlichia chaffeensis
Source: Emerg Microbes Infect. 2021 Mar 13;10(1):461–71. doi: 10.1080/22221751.2021.1899054 (PMC7971322; doi:10.1080/22221751.2021.1899054)

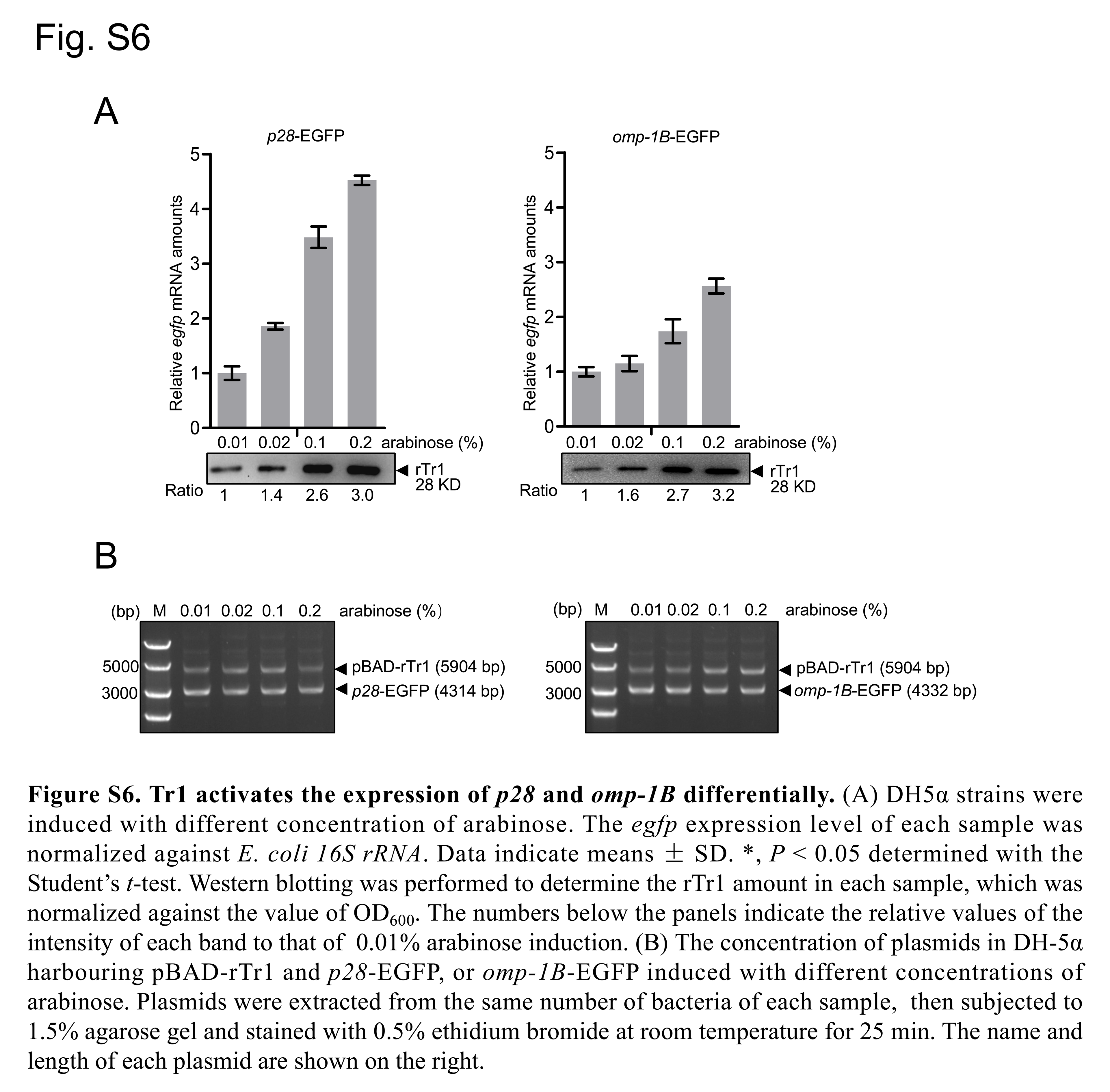

Supplement: fig_s6.tif [file TEMI_A_1899054_SM8601.tif]

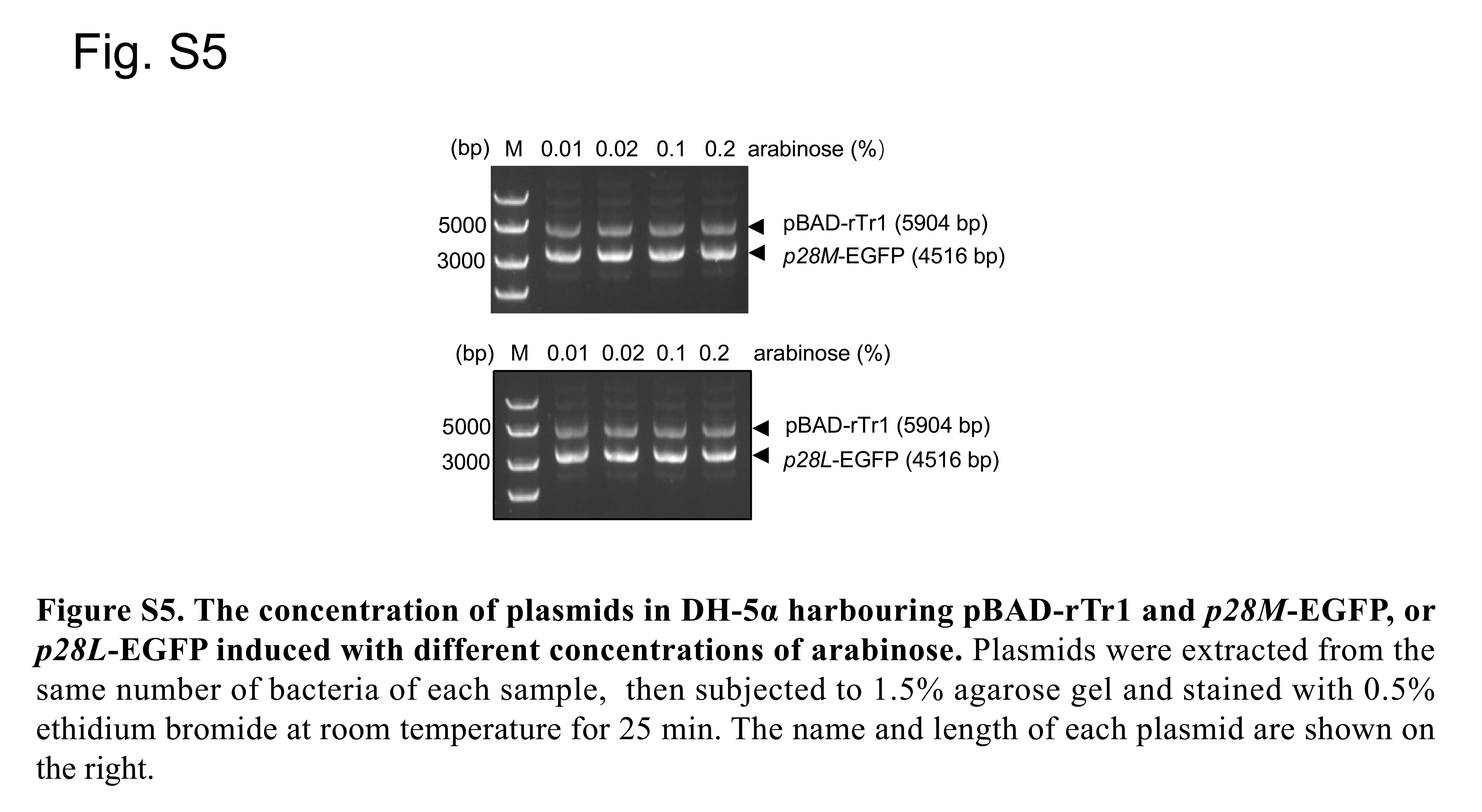

Supplement: fig_s5.tif [file TEMI_A_1899054_SM8600.tif]

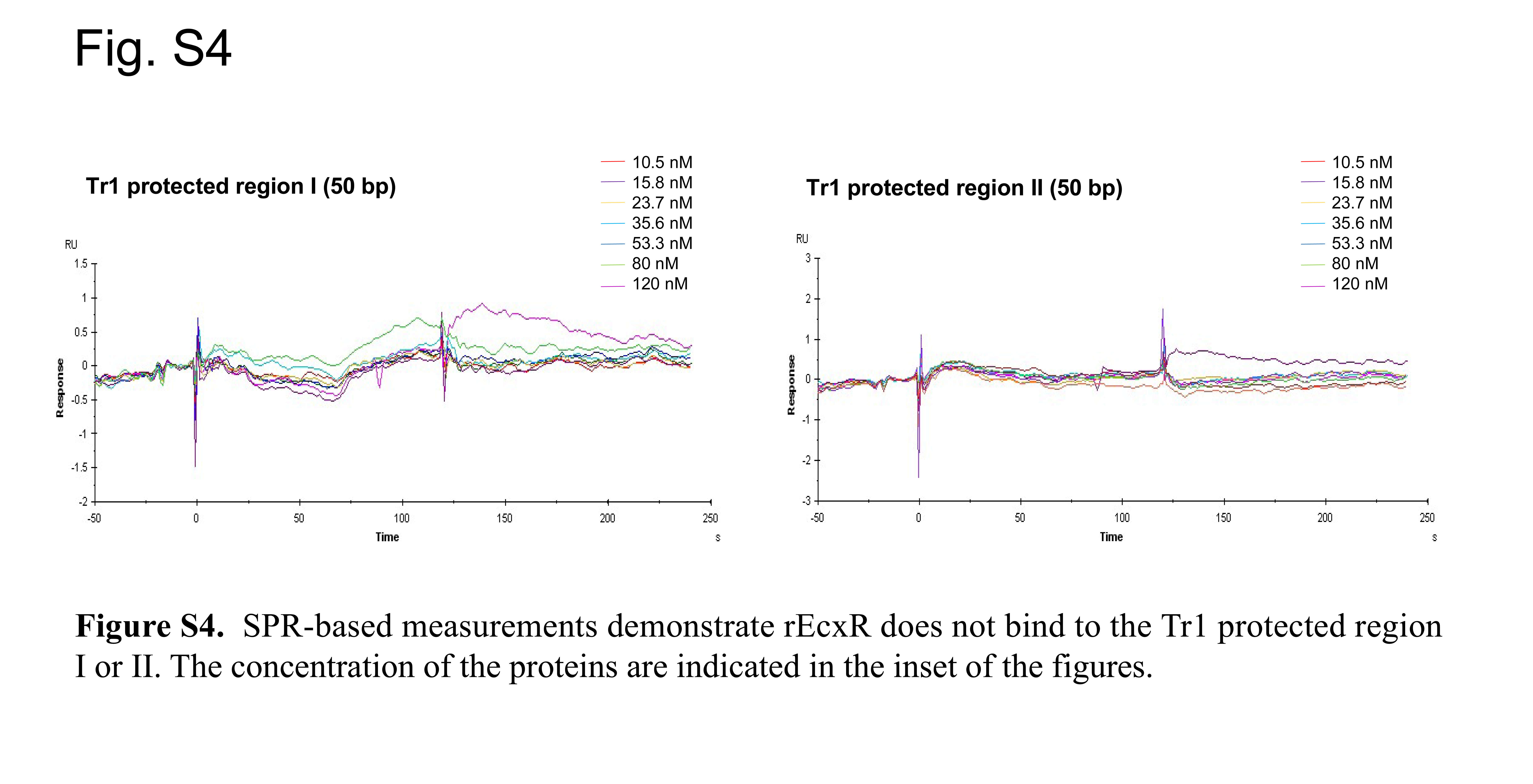

Supplement: fig_s4.tif [file TEMI_A_1899054_SM8599.tif]

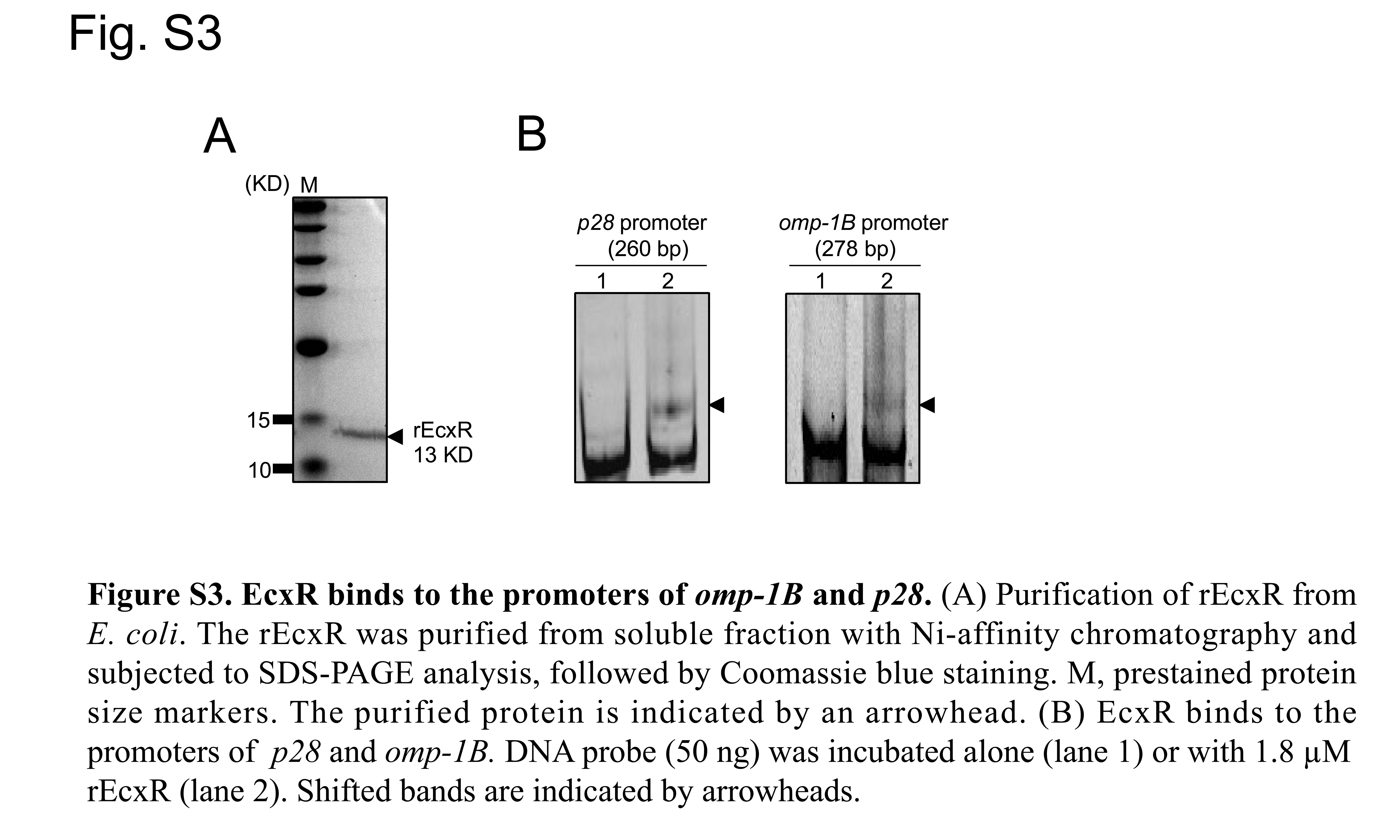

Supplement: fig_s3.tif [file TEMI_A_1899054_SM8598.tif]

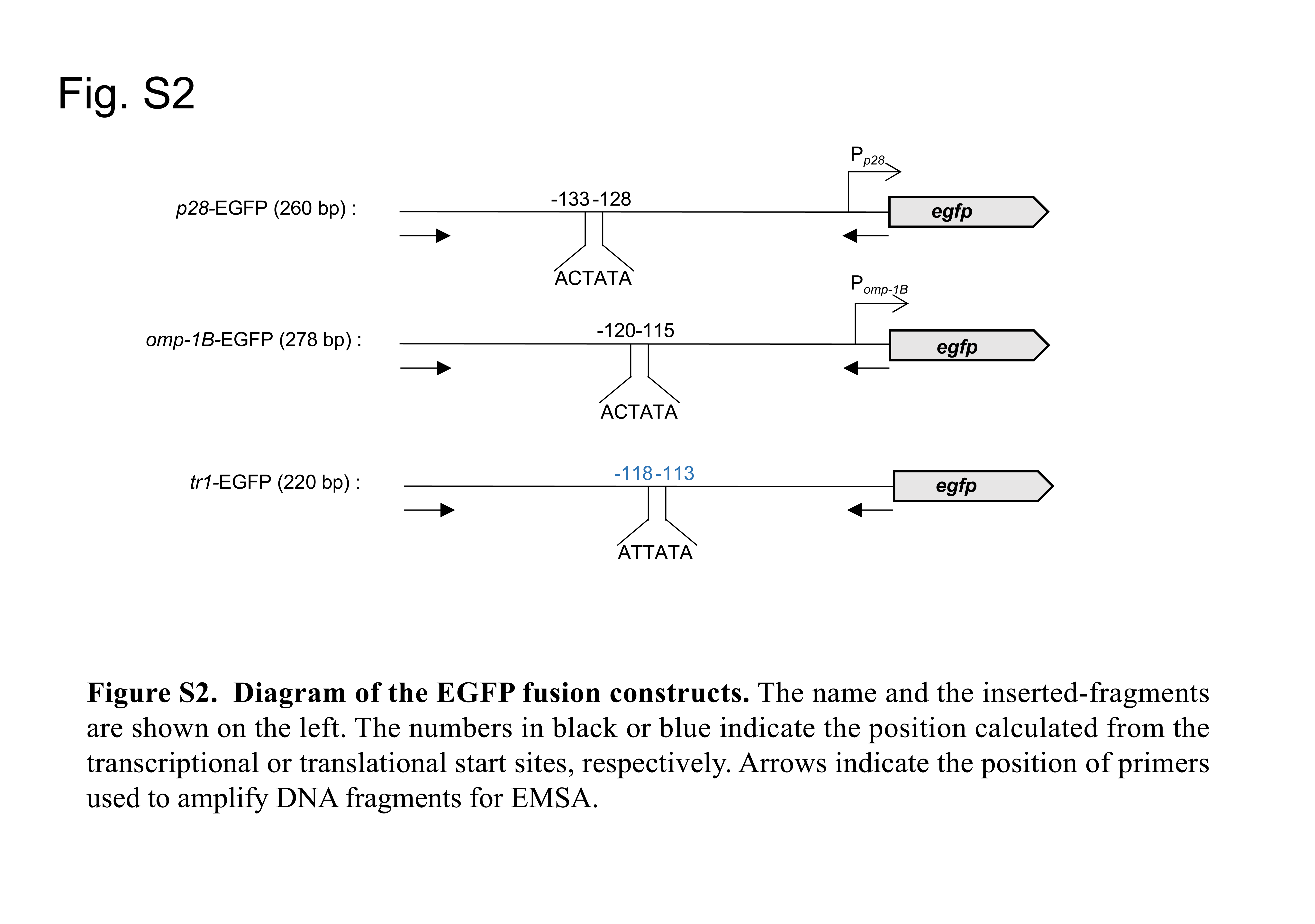

Supplement: fig_s2.tif [file TEMI_A_1899054_SM8597.tif]

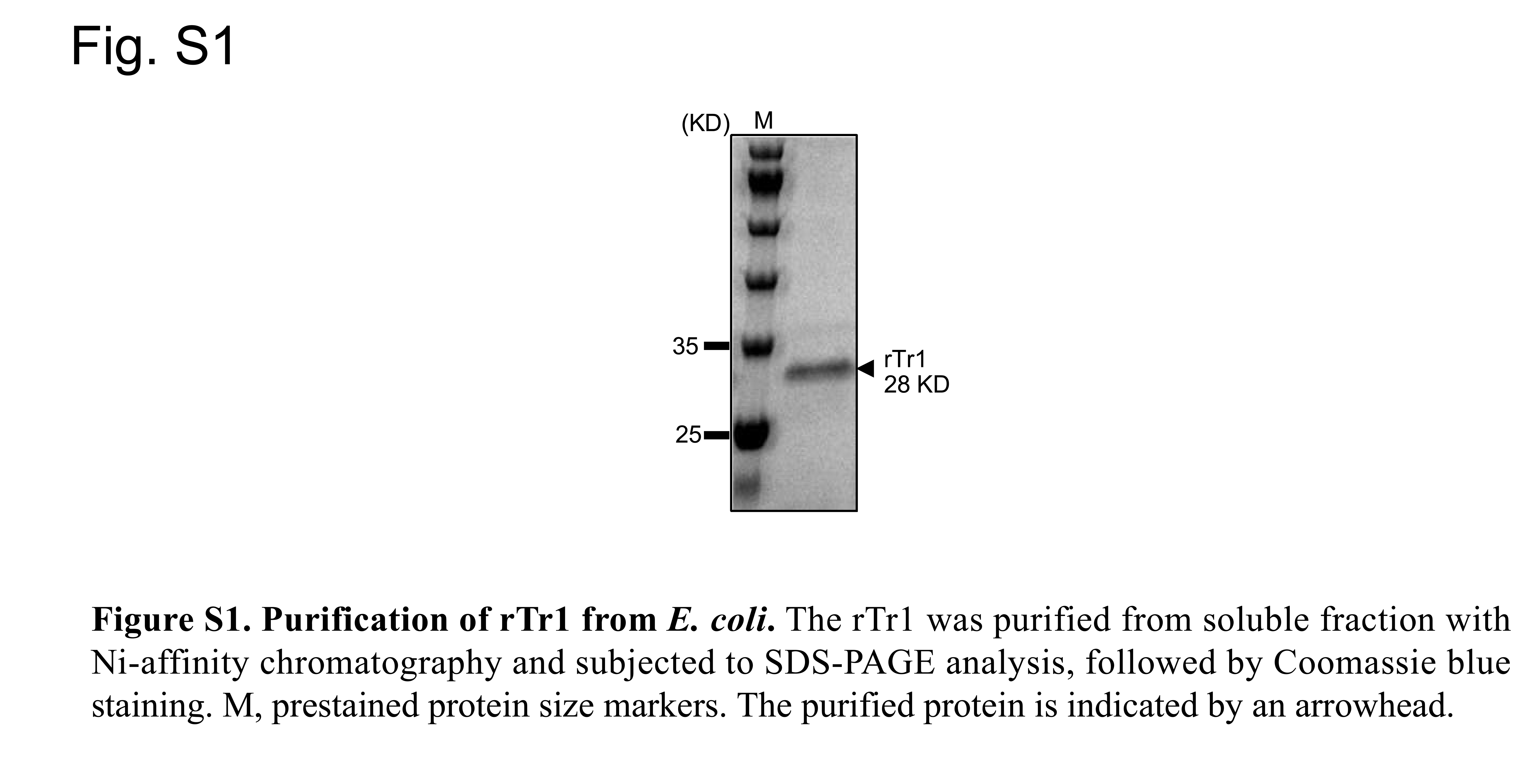

Supplement: fig_s1.tif [file TEMI_A_1899054_SM8596.tif]
